# Molecular Detection of Azole-Resistant *Aspergillus fumigatus* in Clinical Samples

**DOI:** 10.3389/fmicb.2018.00515

**Published:** 2018-03-21

**Authors:** Jochem B. Buil, Jan Zoll, Paul E. Verweij, Willem J. G. Melchers

**Affiliations:** ^1^Department of Medical Microbiology, Radboud University Medical Center, Nijmegen, Netherlands; ^2^Center of Expertise in Mycology Radboudumc/CWZ, Nijmegen, Netherlands

**Keywords:** azole resistance, *Aspergillus fumigatus*, polymerase chain reaction, azole, antifungal, diagnostics

## Abstract

*Aspergillus* diseases are often caused by *Aspergillus fumigatus*. Azoles are the mainstay of therapy, but the management of aspergillosis is hampered by the emergence of azole resistance. Rapid detection of azole resistance might benefit treatment outcome by early treatment modifications. However, the yield of fungal culture in invasive aspergillosis is low and susceptibility testing requires several days to be completed. To overcome the low yield of fungal cultures and slow detection of resistance, it is possible to use molecular tools directly on clinical specimens in order to rapidly detect molecular markers of azole resistance. Molecular tools to detect resistant markers in the *Cyp51A* gene can be expected to be less sensitive compared to molecular tools to detect *Aspergillus* DNA as the *Cyp51A* gene is a single copy gene and the target for *Aspergillus* DNA is often a multi-copy gene. In this mini-review, we summarize the current molecular tools for detection of azole-resistant *A. fumigatus* directly in clinical material. Several in-house PCR assays have been applied directly on clinical material. Furthermore, two assays are commercial available; the AsperGenius and MycoGENIE. The amplification of resistance markers was successful in 70–100% of samples that were positive for *Aspergillus* DNA in BAL samples using the AsperGenius assay. Despite using several samples per patient, amplification of resistance markers was only successful in 33–57% of patients with *Aspergillus* DNA in blood. Furthermore, several sequence based methods have been applied with the benefit of the ability to detect other *Cyp51A* gene alterations.

## Introduction

*Aspergillus* species cause a wide spectrum of disease, including invasive aspergillosis (IA), chronic pulmonary aspergillosis (CPA), *Aspergillus* bronchitis, and allergic bronchopulmonary aspergillosis (ABPA). The most common species involved in aspergillus diseases is *Aspergillus fumigatus*. If antifungal treatment is required, azoles are the first line of treatment, independently of the manifestation of disease (Kosmidis and Denning, 2015). However, the management of aspergillosis is hampered by the emergence of azole resistance (Verweij et al., 2016). Several case series indicate that detection of azole resistance is associated with treatment failure (van der Linden et al., 2011; Chong et al., 2016).

The detection of azole resistance in IA is challenging as the yield of fungal cultures is generally low and phenotypical evaluation of azole susceptibility is therefore often impossible. Furthermore, even if a culture is positive for *A. fumigatus*, susceptibility testing requires several days before results are available. A mature culture is recommended for MIC-testing, which requires up to 7 days of incubation and MICs can be read after an additional 48 h. As early treatment of patients with IA with an appropriate drug improves outcome (Hauggaard et al., 2002), inappropriate therapy due to azole resistance may increase treatment failure. Nowadays it is possible to use molecular tools directly on clinical specimens in order to rapidly detect resistance markers. In this review, we summarize the current molecular tools of azole resistance detection in *A. fumigatus* directly in clinical material.

## Molecular Markers of Azole Resistance in *A. fumigatus*

The hot spot for resistance mutations in *A. fumigatus* is the *Cyp51A* gene, a gene important for the ergosterol biosynthesis pathway. Generally, two groups of resistance mutations can be acknowledged; the environmental resistance mutations and the resistance mutations due to long-term azole therapy. TR_34_/L98H and TR_46_/Y121F/T289A are the most common environmental resistance mutations which are now recovered globally. Furthermore, several less prevalent environmental resistance mutations have been reported including TR_53_, TR_46_^3^, and TR_46_^4^ (three and four repeats of TR_46_, respectively) (Zhang et al., 2017). The other group of resistance mutations consists of point mutations in the *Cyp51A* gene at various positions including G54, M220, P216, G138, and G448 with mutations at locus G54 and M220 leading to the most common amino acid substitutions (Meis et al., 2016; Verweij et al., 2016).

It is estimated that 10% of azole resistance is not *Cyp51A* mediated (Meis et al., 2016). Several other resistance mechanisms have been described including increased efflux pump activity and a substitution in the *HapE* gene (Camps et al., 2012; Fraczek et al., 2013). Moreover, it seems likely that several other, yet uncharacterized, mechanisms are in involved in azole resistance in *A. fumigatus*.

## Molecular Tools to Detect Azole Resistance Directly in Clinical Samples

### Real-Time Resistance-PCR Tests

#### In-House Assays

The first report of the use of a qPCR for the detection of resistance mutations on clinical samples was in 2010 in a case of pulmonary and cerebral aspergillosis (van der Linden et al., 2010). A pulmonary isolate was phenotypically resistant to azoles. Brain biopsy showed septate hyphae but fungal cultures remained negative. Sequence-based analysis of the pulmonary isolates showed the TR_34_/L98H mutation and a real-time PCR was applied on the brain tissue. For detection of the L98H mutation, a 122-bp fragment was amplified, using a hybridization probe to detect the L98H mutation. Furthermore, a second amplification of a 110-bp fragment was used to detect the 34-bp insertion (TR_34_) using a TaqMan probe that was designed to bind to the last 13-bp and the first 9-bp of the 34-bp insertion. The real-time PCR revealed the presence of a L98H mutation as well as the 34-bp insertion (van der Linden et al., 2010). Further details of the published papers evaluating qPCR resistance detection can be found in **Table 1**.

**Table 1:** Overview of studies that applied real-time PCR assays directly on clinical specimen to detect azole resistance markers in *Aspergillus fumigatus.*

Country	Patients samples	Resistance markers	PCR system	Remarks	Positivity	Reference
Netherlands	Tissue (FFPE) from single case	TR_34_, L98H	In-house		1 *Aspergillus* DNA positive 1 *Cyp51A* profiling successful 1 R	van der Linden et al., 2010

United Kingdom	Selection of 29 *Aspergillus* PCR positive sputa	TR_34_, L98H, G54, G138C, M220	In-house	Nested amplification of target with qPCR with allele specific molecular beacons. Primer and probe sequences not published	29 *Aspergillus* DNA positive: 29 *Cyp51A* profiling successful. 27 L98H, 4 M220, 18 TR_34_	Denning et al., 2011

Denmark	94 BAL samples from 87 patients	TR_34_, L98H, G54, G138C, M220, G448S	In-house	Assay described by Denning et al. (2011) with additional molecular beacon for G448S	41 *Aspergillus* DNA positive: 36 *Cyp51A* profiling successful in 44 BAL samples GM > 3. 21 *Aspergillus* DNA positive and 18 *Cyp51A* profiling successful in 32 BAL samples GM 0.5–3. 9 *Aspergillus* DNA positive and 7 *Cyp51A* profiling successful in 18 GM negative BAL samples.	Zhao et al., 2013

Netherlands	BAL	TR_34_, L98H, Y121F, T289A	AsperGenius	LoD species at <36 *C*t	16 *Aspergillus* DNA (<36 *C*t) positive. 14 Cyp51A profiling successful 12 WT 2 R	Chong et al., 2015

United Kingdom	Serum	TR_34_, L98H, Y121F, T289A	AsperGenius	LoD of species and resistance assay was 10 and >75 genomes	25 *Aspergillus* DNA positive, 12 *Cyp51A* profiling successful 5 WT/7 R	White et al., 2015b

Netherlands	BAL	TR_34_, L98H, Y121F, T289A	AsperGenius	LoD species at <38 *C*t	97 *Aspergillus* DNA positive (<38 *C*t) 68 *Cyp51A* profiling successful 57 WT/11R	Chong et al., 2016

Belgium	BAL samples of which 45 GM positive	TR_34_, L98H, Y121F, T289A	AsperGenius	LoD species at <36 *C*t	20 *A. fumigatus* DNA positive (<36 Ct) 20 *Cyp51A* profiling successful 17WT/3 R	Montesinos et al., 2017

United Kingdom	Plasma of 12 patients	TR_34_, L98H, Y121F, T289A	AsperGenius	LoD of species and resistance assay was 10 and 50 genomes. Meanly 7 samples per patient.	6 patients *A. fumigatus* DNA positive, 2 *Cyp51A* profiling successful 2 WT	White et al., 2017

France	Respiratory + Serum	TR_34_, L98H	MycoGENIE	LoD of species and resistance assay was 1 and 6 genomes.	Respiratory: 55 *A. fumigatus* DNA positive Serum: 16 *A. fumigatus* DNA positive 0 Cyp51A R	Dannaoui et al., 2017

France	Respiratory	TR_34_	MycoGENIE	No WT *Cyp51A* gene – control in assay.	106/136 *A. fumigatus* DNA positive. 0 *Cyp51A* R.	Morio et al., 2018

Netherlands	Respiratory samples. 91 samples tested. Three cases with co-infection described	TR_34_, L98H, Y121F, T289A	AsperGenius	In one case both resistant and susceptible isolates cultured. No sequence information of isolates available.	72 *Aspergillus* DNA positive, 45 *Cyp51A* profiling successful 3 samples showed presence of both WT and resistant peaks	Schauwvlieghe et al., 2017

*FFPE, formalin-fixed paraffin-embedded; BAL, bronchoalveolar lavage; LoD, limit of detection.*

Another in-house PCR assay was used on 29 *Aspergillus* DNA positive sputum samples from patients with CPA and ABPA. In this series a nested-PCR approach was used. The *Cyp51A* gene was amplified in two fractions and a qPCR with allele-specific molecular beacons was used to detect mutations at locus G54, L98, G138, and M220 and to detect the 34-bp insertion. *Cyp51A* was successfully amplified in all 29 *Aspergillus* PCR positive samples. Four samples had a mutation at M220, 27 of 29 had a L98H mutation of which 16 also had TR_34_. TR_34_ without L98H was found in two patients. In two patients with a M220R mutation, TR_34_ and L98H were also detected. Only four samples were culture positive, in three the molecular results did not correspond with the culture results (Denning et al., 2011). The detection of either TR_34_ or L98H alone and a TR_34_/L98H with an additional M220 mutation has not been reported in cultured isolates, to the best of our knowledge. It seems likely that due to fact that two genomic regions are amplificated independently for the detection of TR_34_ and L98H, the amplicons were from different strains, (e.g., a susceptible and resistant strain), resulting in the single detection of either TR_34_ or L98H. The frequency of resistance markers seems very high in this series and these results have not yet been confirmed by other studies.

The same nested PCR-assay, with the addition of a new molecular beacon for G448, was used to analyze 94 BAL samples. Sixty-one of 71 (86%) samples positive for a pan-*Aspergillus* PCR had a successful amplification of the *Cyp51A* gene. Four of the *Cyp51A* negative samples were culture positive for *A. flavus*. One sample contained M220V and another P216L. The authors did not describe the use of a molecular beacon for P216L and the mutation was thus presumably detected by subsequent sequence analysis (Zhao et al., 2013). The high rate of TR_34_/L98H mutations observed by Denning et al. (2011) was not observed in the latter study from Denmark.

#### Commercial Available Assays

Results from the first commercial available resistance PCR assay, the AsperGenius (PathoNostics, Maastricht, Netherlands) were published in 2015. The qPCR has the ability to detect *Aspergillus* DNA, and differentiates *A. fumigatus* and *A. terreus*. In addition, four mutations (TR_34_ and L98H to detect TR_34_/L98H, Y121F, and T289A to detect TR_46_/Y121F/T289A) can be detected using melting-curve analyses. The PCR was validated on stored BAL fluid samples from hematological and ICU patients. Of 19 BAL fluids, 16 were positive for *Aspergillus* DNA, whereas 14 of 16 were positive for *A. fumigatus*. All 14 samples were also positive for resistance targets and mutation analyses revealed 12 samples with WT *Cyp51A* DNA, one TR_34_/L98H and one TR_46_/Y121F/T289A (Chong et al., 2015).

The assay was validated prospectively in a multicenter study in 201 patients with hematological disease suspected for IA. The *Aspergillus* PCR was positive in 97 samples (74 samples with either galactomannan (GM)-index >1 or with a positive culture) and the resistance PCR was successful in 68 of 97 (70%) samples. In 7 samples TR_34_/L98H was detected, in one sample TR_46_/Y121F/T289A, while a combination of TR_34_/L98H and WT *A. fumigatus* was observed in three samples. Importantly, this study showed that the detection of resistance markers was associated with azole treatment failure; even in culture-negative cases (Chong et al., 2016).

Another study used the assay on 45 GM-positive BAL samples and 55 GM-negative BAL samples. Twenty-seven of 100 patients were positive for *Aspergillus* DNA and 20 for *A. fumigatus* DNA. The amplification of resistance markers appeared to be successful in all *A. fumigatus* positive samples and evidence for azole resistance was found in three (Montesinos et al., 2017).

Other studies have evaluated the AsperGenius assay directly on blood samples, for early detection of *Aspergillus* disease in patients at risk for IA (White et al., 2015a). The assay was evaluated on serum samples from 14 patients with proven or probable IA (total 54 extracts). *A. fumigatus* DNA was detected in 25 of 54 samples. The amount of DNA in the serum, however, was low and only 12 samples of 7 cases had sufficient DNA-levels for the resistance PCR to be performed reflecting the lower sensitivity of the resistance PCR compared to the *Aspergillus* DNA assay. Samples from four patients (57%) had 3–4 loci (TR_34_, L98H, T289A, or Y121F) amplified and 50% of all patients had at least one loci amplified. Mutation analyses showed no mutations in these patients. Thus despite using multiple samples per patient; genotyping both TR_34_/L98H and TR_46_/Y121F/T289A was possible in only four of 14 patients with proven/possible IA. For successful amplification of resistance markers, a *C*t value of <33 was needed in the *A. fumigatus* assay (White et al., 2015b).

As the performance of *Aspergillus*-PCR was shown to be superior on plasma compared to serum, it was hypothesized that plasma was also superior for the detection of resistance markers (White et al., 2015a). Eighty-six plasma samples from 10 patients with proven/probable IA and two patients with possible IA were tested. Eleven samples from six patients were positive for *A. fumigatus*. Amplification of resistance markers was successful in only two of six (33%) patients. Thus, using a mean of seven samples per patient, the resistance markers were successfully amplified in only two of 12 (17%) patients with IA. However, as the *A. fumigatus* PCR was only positive in six of 12 patients and all culture results were negative, it is not known whether the IA was due to *A. fumigatus* in the other patients (White et al., 2017). As the target for azole resistance detection is a single copy gene, the sensitivity can be expected to be lower than the *Aspergillus* DNA assay for which a multi-copy gene is targeted. The results of PCR used on blood samples clearly reflects this lower sensitivity and due to the low amount of DNA in blood samples, clinical utility in routine diagnostics is questionable.

An interesting advantage of the AsperGenius PCR is the simultaneous detection of both WT and mutant *Cyp51A* DNA. As the detection of resistance markers is based on melting curve analysis, mixed infections with both WT as well as DNA from resistant strains will result in two peaks in the assay. This was shown in three cases were evidence for both WT and L98H was found (Schauwvlieghe et al., 2017).

A report of a second commercial available assay, the MycoGENIE (Ademtech, Pessac, France), was published in 2017. The MycoGENIE is a multiplex assay detecting *A. fumigatus* DNA, the L98H mutation and TR_34_. The PCR was tested on a total of 55 serum samples from 16 patients and 88 respiratory samples from 62 patients. Fifty-Five respiratory and 16 serum samples were positive for *A. fumigatus* (Dannaoui et al., 2017). The same MycoGENIE was then used on stored clinical samples from 137 patients with fungal rhinosinusitis with a positive microscopy for fungal hyphae. The PCR was positive for *A. fumigatus* in 116 of 147 samples (78.9%), whereas 47 of 48 *A. fumigatus* culture positive samples were positive in the assay (Morio et al., 2018). In both studies no samples were positive for either TR_34_ or L98H.

An important limitation of the MycoGENIE is the absence of WT probes for the resistance marker. As a consequence, non-positivity in the resistance markers might be caused a WT genotype but might also be due to a missed mutation due to limited sensitivity compared to the probe for detection of *A. fumigatus* DNA. Furthermore, the less prevalent environmental TR_46_/Y121F/T289A mutation is not detected by the MycoGENIE.

### Sequence Based Assays

In addition to direct qPCR assays, several other methods have been applied directly to clinical specimen (**Table 2**). Amplification with subsequent pyrosequencing was applied directly on blood samples to detect G54 mutations in the *Cyp51A* gene. *A. fumigatus* DNA was detected in two of 56 whole-blood samples targeting a 269-p *Cyp51A* gene region and *Cyp51A* sequence of locus G54 was determined. The study showed the utility of amplification and subsequent sequencing of clinical material for detection of *Cyp51A-*mutations (Trama et al., 2005). Most other loci were not covered by the targeted region but this can be overcome by sequencing of additional *Cyp51A* regions involved in azole resistance.

**Table 2:** Overview of studies that applied non-real-time molecular tools directly on clinical specimen to detect azole resistance markers in *A. fumigatus*.

Country	Patients samples	Resistance markers	PCR system	Remarks	Positivity	Reference
United States	56 whole blood samples	G54	Pyrosequencing	No control was available for sequence results	2 *Cyp51A* DNA positive 2 *Cyp51A* profiling successful 2 WT	Trama et al., 2005

Germany	BAL fluid and tissue samples from 8 patients positive for *A. fumigatus* DNA	TR_34_, L98H, M220	Sequencing		8 *A. fumigatus* DNA positive 8 *Cyp51A* profiling successful	Spiess et al., 2012

Germany	Serum, BAL, tissue from 155 patients	TR_34_/L98H/TR_46_/M220	TR34 M220 TR46 nested sequenced based	LoD: L98H, 300fg. TR46, 300fg. Other assays: Spiess et al. (2012)	181 *Aspergillus* DNA positive, 26 L98H profiling successful, 17 L98H and TR_34_ profiling successful, 32 L98H, TR_34_ and TR_46_ profiling successful	Spiess et al., 2014

France	97 respiratory samples	TR_34_, L98H, G54, G138C, M220	Sequencing		44 *Aspergillus* DNA positive, 38 *Cyp51A* profiling successful	Zhao et al., 2016

*LoD, limit of detection.*

Other authors generated additional primers for the specific amplification and subsequent sequencing of the *A. fumigatus Cyp51A-*gene. Three different primer sets were used for the amplification of the *Cyp51A* promoter region, L98 and M220 regions. Eight stored clinical samples were tested and TR_34_/L98H was successfully detected in a brain abscess. Interestingly, in one BAL fluid, L98H was found without the TR_34_ (Spiess et al., 2012). Using the same method with the addition of another target region for the detection of TR_46,_ stored clinical samples of 155 immunocompromised patients previously tested positive for *Aspergillus* DNA were evaluated. In 75 of 181 (41%) investigated samples, the assay was positive for at least one region. The L98H region alone was successfully amplified and sequenced in 26 (14%), the regions for L98H and TR_34_ in 17 (9%); both revealed no mutations. In 22 of 181 (12%) samples the L98H, TR_34_ as well as the M220 PCR targets were successfully amplificated, showing two samples with both the TR_34_ and L98H mutations. One sample had L98H but not TR_34_ (Spiess et al., 2014). Higher rate of successful amplification was achieved by a nested approach. The sequence method described by Denning et al. (2011) was used but without the molecular beacons. The *Cyp51A* was successfully amplified in 38 of 44 (86%) pan-*Aspergillus* PCR positive samples. Using this technique, the authors found a sputum sample harboring TR_34_/L98H, which was confirmed by culture (Zhao et al., 2016).

## Conclusion and Perspectives

The application of molecular tools has been shown to increase the sensitivity of detection of azole resistance in *A. fumigatus* compared to culture. Furthermore, the detection of resistance markers in BAL samples using the AsperGenius was associated with increased probability of azole treatment failure. The commercial available assays target TR_34_/L98H and TR_46_/Y121F/T289A mutations and are able to detect most of azole-resistant *A. fumigatus* in patients with acute IA. However, the clinical utility depends on regional resistance epidemiology. In patients with prolonged azole therapy, a greater diversity of resistance mutations should be taken into consideration. Detection of resistance markers in blood samples remains problematic; nonetheless if very low *C*t value in the *Aspergillus* PCR is observed, resistance markers might be successfully detected.

Direct amplification and subsequent sequencing of the *Cyp51A-*gene showed the potential to cover most *Cyp51A*-mediated resistance mutations. However, compared to real-time assays, sequencing is relatively time-consuming, expensive and laborious, and might lack sensitivity due to low amounts of DNA in most clinical samples. Furthermore, compared to the commercial qPCR assay for detection of azole resistance, clinical validation studies are currently lacking.

New molecular assays should aim to increase sensitivity and extend the coverage of resistance markers to include both environmental as other azole resistance mutations. Sequencers have become portable, inexpensive and can be used in real time. When the capacity of whole genome sequencing increases in the near future, it seems likely that whole genome sequencing can be applied directly on clinical specimens to detect not only the pathogen, but also a wide range of resistance mechanisms.

## Author Contributions

WM, JZ, and JB reviewed the available papers. JB wrote the first draft manuscript. WM and PV edited the manuscript. All authors gave their final approval.

## Conflict of Interest Statement

PV has received research grants from Gilead Sciences, Astellas, Merck Sharp & Dohme (MSD), F2G, and BioRad, is a speaker for Gilead Sciences and MSD, and is on the advisory boards for Pfizer, MSD, and F2G. The other authors declare that the research was conducted in the absence of any commercial or financial relationships that could be construed as a potential conflict of interest.
